# Editorial

**Published:** 1860-10

**Authors:** 


### EDITORIAL.

*Legislation on Medical Subjects.*—We would call the special attention of our readers to the Report of the Medical Society of Galesburg, contained in the present number of this journal. The subjects there brought up for consideration are very important, both to the profession and the people of the State. If the statutes of this State actually require a physician, when called into court, as a witness, to divulge anything that may have been told him by a patient, and which was strictly necessary for the proper treatment of such patient, they are contrary to the plainest dictates of justice, and ought certainly to be altered. The advantages to be derived from the enactment and enforcement, of a plain and practical law for registering births, marriages, and deaths, throughout the State, are too obvious to the medical reader to require elucidation. The statistics thus derived afford us the only reliable data, for determining the influence of locality, climate, etc., on the prevalence of particular diseases, their fatality, and the comparative longevity of the people. And, yet, these are all topics intimately connected with the welfare and happiness of the whole community. That it is the duty of the Legislature, to make some provision by which the study of human Anatomy can be prosecuted without the violation of law, is equally apparent to every reflecting mind. This topic the reader will find more fully discussed in the Annual Address to the State Medical

Society, published in the *Examiner* for June, 1860. We would urge all the local Medical Societies throughout the State, to actively co-operate in bringing these topics before the next Legislature. And we would suggest that each practitioner use his influence, personally, with the candidates for the next Legislature *before* the election day in November.

---

*Medical Department of Lind University.*—The Second Annual Course of Lectures in this institution, commences on Monday, the 8th inst. The general Introductory Lecture will be given by Prof. Byford, in the College Hall, No. 22, Market street, at 7½ o'clock in the evening. The Trustees of the University, the Professors in other departments, the members of the Medical Profession, and the public generally, (including the ladies,) are invited to attend. After the Lecture the Museum and Library of the College will be open for the examination of the public.

---

*Chicago City Dispensary.*—This charitable institution was first opened some three years since, on west Randolph street, by Drs. Wardner, Andrews, and Hollister. About the 1st of September, 1859, it was removed to No. 22 Market street, and placed under the immediate charge of Professors Andrews and Davis, in connection with the University Medical College. During the year just passed, ending Sept. 1st, 1860, over 2000 poor patients have received advice and medicine gratuitously, and several lying-in women, too poor to pay for medical services, have been furnished with reliable medical attendance at their homes. During the months of July and August, while diarrhœas were prevalent among children, the daily attendance of patients was between 20 and 30, two-thirds of whom were infants and young children. It will thus be seen that this charity is one of the most extensive and important in the city. Neither are its benefits restricted to the poor patients alone; for throughout the whole year, one Medical and one Surgical Clinic, has been given each week, to such Medical Students as were pursuing their studies in the city. And there is no place in the country that affords a better opportunity for instruction in diseases af children, than in this Dispensary during the summer months. Hereafter Dr. Byford, Professor of Obstetrics and Diseases of Women and Children, will take the place of Prof. Davis in the Dispensary, and give Clinical instruction especially on diseases of Women and Children every Saturday afternoon; Prof. Andrews still retaining the Surgical department as heretofore. Medical attendance will also be supplied to lying-in women, at their homes, (if too poor to pay for medical services,) by applying at the Dispensary at the usual hours, between 2 and 3 o'clock in the afternoon of each day except Sundays.

---

### MERCY HOSPITAL REPORT.

During the year ending August 1st, 1860, there were admitted into the Hospital of the Sisters of Mercy, 282 patients; of whom 204 were admitted into the Medical Wards, and 78 into the Surgical. Of those received into the Medical Wards, the principal diseases and results were as follows:

| | Cases. | Recovered. | Improved. | Died. |
|---|---|---|---|---|
| Typhoid Fever,.......... | 16 | 15 | 0 | 1 |
| Typhus Fever,.......... | 2 | 2 | 0 | 0 |
| Intermittent Fever, ...... | 10 | 10 | 0 | 0 |
| Remittent Fever, ........ | 11 | 11 | 0 | 0 |
| Dysentery,............... | 11 | 11 | 0 | 0 |
| Diarrhœa, chronic ulcera'n, | 10 | 5 | 3 | 2 |
| Pneumonia,............. | 8 | 7 | 0 | 1 |
| Bronchitis, chronic, ...... | 4 | 2 | 2 | 0 |
| Rheumatism,............ | 10 | 9 | 1 | 0 |
| Delirium Tremens,....... | 12 | 9 | 2 | 1 |
| Pulmonary Consumption,.. | 8 | 1 | 5 | 2 |
| Scarlet Fever,.......... | 2 | 2 | 0 | 0 |
| Scirrhosis with Ascites, ... | 1 | 0 | 1 | 0 |
| Fatty Liver with dropsy,.. | 2 | 0 | 0 | 2 |

The remaining 97 cases embraced a large variety of diseases, both acute and chronic, among which the different varieties of opthalmia and cutaneous eruptions were the most numerous. It will be seen that the ratio of deaths to the whole number of cases treated, is 1 in $22\frac{2}{3}$. The Hospital is under the charge of Prof E. Andrews, in the Surgical department, and Prof. N. S. Davis, in the Medical. During the Lecture season of the Medical Colleges of this city, Clinical instruction is given in the wards of the Hospital from 8 to 9 o'clock every week-day morning, and three mornings per week all the rest of the year; thus constituting it a continuous school of practical instruction. The price of Tickets for admission to the Clinical instruction is $6.00, and the Ticket is good for the whole year. Patients are received and treated, both in the Medical and Surgical wards, from any part of the country, on the payment of from $3 to $5 per week for their board, according to the ward they occupy.

---

### PUFFING.

"The *Reporter* has become *the leading Medical Periodical of America.* It has attained this position by representing the *whole* Profession, independent of any school, party, or publishing interest, *and will maintain it.*"

The above, is one of the paragraphs kept at the head of the editorial columns of the *Medical and Surgical Reporter:* a weekly journal, published in the same city with the old *Quarterly Journal of Medical Sciences*, and the more recent *North-American Medico-Chirurgical Review.* We would respectfully ask the highly esteemed editors of the *Reporter* the following questions:

1st, What is the difference in principle, between claiming to publish "the leading Medical periodical of America," and claiming to be *the leading* practitioner of Medicine of America?"

2nd, In what respect does the *Reporter* represent the "whole Profession?"

3rd, How is it any more "*independent*," than a dozzen other Medical journals in this country?

4th, Would it not be more modest as well as more appropriate, to wait for our contemporaries and posterity to assign us and our works their proper position, than to be continually puffing ourselves?

### NEW YORK MEDICAL COLLEGE AND CHARITY HOSPITAL.

Under this title the New York Medical College, has been re-organized. One-third of the college building has been set apart for a Hospital, and the number of Professorships has been increased to ten. The following constitute the present Faculty:

R. Ogden Doremus, M.D., Professor of Chemistry.

J. M. Carnochan, M.D., Professor of Clinical and Operative Surgery.

D. Meredith Reese, M.D., L.L.D., Professor of Theory and Practice of Medicine, and Medical Jurisprudence.

B. J. Raphael, M.D., Professor of Principle and Practice of Surgery, and of Surgical Pathology.

A. K. Gardner, M.D., Professor of Clinical Midwifery and Diseases of Females.

John O. Bronson, M.D., Professor of Anatomy.

Chas. A. Budd, M.D., Professor of Theory and Practice of Midwifery.

A. Jacobi, M.D., Professor of Infantile Pathology and Therapeutics.

Bern. L. Budd, M.D., Professor of Toxicology.

R. K. Brown, M.D., Professor of Physiology.

M. Bailey, M.D., Adjunct Professor of Anatomy.

Fowler Prentice, M.D., Demonstrator of Anatomy.

The regular Lecture term continues five months, viz.: from the 17th of October to the 17th of March. It does not appear, that with this large number of Professors, they either divide the annual term of instruction into two departments, or materially increase the gross amount of instruction given during the term.

In the faculty we recognise, however, the names of several men noted for their ability and zeal, in advocating a higher standard of Medical education; and we must look with confidence for them to practise what they *preach.*

### MEDICAL DEPARTMENT OF UNIVERSITY OF MICHIGAN.

Samuel Denton, M.D., Professor of Theory and Practice of Medicine, in this School, died at Ann Arbor, Michigan, Aug. 17th, 1860. Dr. Denton had been connected with the Med. Department of the University from its first organization; and was a man highly esteemed both by the Profession and the community in which he lived.

At a meeting of the Board of Regents held at Detroit, Sept. 14th, the following Resolutions were adopted:—

"*Resolved*, That Prof. A. B. Palmer be appointed Professor of the Theory and Practice of Medicine, of Pathology, and of Materia Medica, with a salary of one thousand dollars.

"*Resolved*, That Prof. Moses Gunn be appointed Professor of Surgery and Therapeutics, with the same salary.

"*Resolved*, That Prof. Abram Sager be appointed Professor of Obstetrics and Diseases of Women and Children, with the same salary.

"*Resolved*, That Prof. Corydon L. Ford be appointed Professor of Anatomy and Physiology, with the same salary."

This reduces the number of Professors, in that school, to *five;* and we believe it is the design to increase the length of the term to *nine* months, thereby making it in all respects much like the Med. Dept. of the University of Virginia, at Charlottesville, in that State.

### EPILEPSY.

Dr. W. M. Connell, in the Charleston *Medical Journal* for July, recommends the following formula in the treatment of Epilepsy:—

℞. Spiritus vini. gallici, Oj.<br>

Tinct. Stromonii Dat., ℥ iv.<br>

Sulph. Zinci, ʒ ii.<br>

Solv. zinc. in aquam distil. ℥ i.

Mix. Give 10 drops at a dose, and increase gradually until the specific effects of the stramonium are induced slightly, and continue it at that point.

### PHYSIOLOGY AND PATHOLOGY OF THE SPLEEN.

Dr. David Hutchison, of Mooresville, Ind., concludes an essay on this subject, published in the Cleveland *Medical Gazette*, for September, with the following propositions :—

"1st. The spleen serves as a diverticulum or reservoir, for the blood, and thus regulates the quantity of the blood in the circulation.

"2nd. The spleen performs a very important office in the process of sanguification, either as a blood-disentegrating or as a blood-forming organ.

"3rd. Malaria acts on the blood, breaking down the blood-corpuscles, from which results disease of the spleen, and a peculiar anemic condition of the whole system.

"4th. The cure of this spleenic affection depends on the administration of Quinine and Iron—especially the latter."

---

*Medical College of Ohio.*—This institution has been reorganised by the appointment of a Faculty, containing only two of those who constituted the previous Faculty. The new appointments appear to be far from satisfactory to the Profession in Cincinnati.

---

### YEAR BOOK OF AMERICAN CONTRIBUTIONS TO MEDICAL SCIENCE AND LITERATURE.

It is designed that part *first* of each volume, shall comprise an arranged and classified *summary* of, and index to, all the important and original papers found in the various *Medical Journals* of this country, for the year immediately preceding. Part *second* will comprise a *summary* of, and index to, all papers found in the published transactions of the National and the various State and County Medical Societies. Part *third* will embrace reviews of Medical books of American authorship, published during the year, with a *summary* of all the novelties in opinion or practice therein.

To the above plan and arrangement, such other additions shall be made as time and circumstances may suggest. The first volume will be issued early in the spring of 1861.

In the preparation of our *Summary of American Medical Journalism*, for the *American Medical Monthly*, we have solicited a copy of all Medical Journals published in this country; the *American Jouanal of Medical Sciences*, the *N. O. Medical and Surgical Journal*, and the *Ohio Medical and Surgical Journal* are the only ones that have failed to comply with the request. To facilitate our design, we request an *exchange* with *all* American Medical Journals, to be sent to our address as issued. All Medical Societies who publish their transactions, will, we trust, be kind enough to send their transactions to us. Publishers of Medical Books, particularly of American authorship, are earnestly requested to send, as soon as issued, *all books* of the character as above.

The importance of a work of the character as above, for the information of the profession, and for the honor and dignity of American Medicine, will readily be conceded by all. We cannot prepare the work and publish at a pecuniary loss, and, hence, the object of this circular is to request that all physicians who would encourage the work and become subscribers to the same, would send us their names *at once*—payment to be made only on publication of the work. The work shall contain from 500 to 1,000 pages, be substantially bound, and furnished at the low price of *three dollars*. That we may know whether the work is to receive sufficient encouragement to justify its completion and publication, we request that subscribers' names may be sent in immediately. As a special favor and encouragement of this truly national enterprise, we would request that all Medical Journals of this country would copy our circular.

To Editors and Publishers we would say that it is designed that our Year Book shall commence its gleanings with the year 1860. Journal editors and book publishers will remember this, in sending their respective publications to our address.

All Books, Journals, published Transactions, and names of Subscribers, should be directed to

O. C. GIBBS, M.D.,

*Frewsburg, Chautauque Co., N. Y.*

*London Lancet.*—The October number is promptly on hand, filled with its usual amount of interesting and valuable matter.

---

*The Cause of Death.*—Out of 100 deaths in England and Wales in 1858, the last year for which the cause of death have been examined, 25 were from zymotic diseases, 19 from constitutional diseases, 37 from local diseases, 16 from developmental diseases, and 3 from accidental or other violence. Zymotic diseases were exceedingly fatal, especially scarlatina, which, with its auxiliary diphtheria, caused 30,317 deaths. Small-pox and measles destroyed—the one 6,460 lives, the other 9,271. Syphilitic diseases killed 1006 persons, above 700 of them infants, who receive it as their only inheritance. Want was recorded as the cause of death in 62 instances; but, observes Dr. Farr, in how many more was it the real, though unavowed, source, or support of fatal disease, it was impossible that register-books could reveal. Almost 1000 children died from want of breast-milk; "alcoholism" destroyed 712 persons, the deaths of 288 being expressly referred to intemperance, and 424 more vaugely to delirium tremens. In the second class,—the constitutional,—which includes tubercular diseases, it is found that the rate of mortality from phthisis in London and in the Welsh division was nearly the same, though the two districts differ widely in important circumstances; but other pulmonary diseases—bronchitis, pneumonia, asthma, &c., were more than three times as fatal in London as in Wales. In the third class—local diseases—there was a clear increase in affections of the brain, the heart, the lungs, and the kidneys, a very remarkable decrease in phlegmon. In the fourth class—developmental diseases—there was an increase in the deaths from old age, caused by the cold of winter. 3,131 mothers died from child bearing—a considerable increase of mortality, supposed to be caused partly by the general unhealthiness of the year, and partly by privations occasioned by the distress resulting from the commercial crisis at the close of 1857. There were six diseases, each of which killed above 25,000 persons in the year—phthisis, 50,442; scarlatina, 30,317; bronchitis, 29,093; atrophy and debility, 26,360; pneumonia,

26,486; convulsions, 25,488 (children). Diseases are ranged in the Registrar-General's reports in 112 classes, or we might say groups, so many are the foes ever on the watch for us. Of the deaths in 1858, half were of persons under seventeen years of age; four out of ten were under five years of age. On the registers for the first quarter of the year being examined, it was found that 7,275 persons died without any medical attendant to certify the cause of their death—six in 100 of the deaths. In Manchester, 181 persons out of 1,755, the number who died in the quarter, had no medical attendance in their last illness; in Yorkshire, as many as 10 persons out of 100, and in the Welsh division at least 12 out of the same number. —*London Lancet.*

---

*London and its Health.*—London, says the Registrar-General, now covers 121 square miles—a square mile of 11 miles to the side. It is equal to three Londons of 1800. It increases at the rate of 1000 a week, half by births (their excess over deaths,) and half by immigration (its excess over emigration.) It is remarkable that in London one of six of those who leave the world dies in one of the public institutions—a workhouse, hospital, asylum, or prison. Nearly one in eleven of the deaths is in a workhouse. For the improvement of the health of London three things are to be aimed at: pure air to breathe, pure water to drink, and a healthy soil to live on. The Registrar-General observes that there are above 2000 medical men in London and its vicinity; but they are chiefly employed in treating disease—the art of preventing it is not cultivated; it is not taught in any of our medical schools; it is not formally the subject of examination in our universities. The father of a family does not go to a doctor and say, "How can I preserve my health, make my children well and vigorous, and develope all their faculties to the fullest extent?" Imagine the 2000 members of the most enlightened profession in the country employed in instructing the people in the way of a healthy life. How many thousands of lives would be saved every year in London! How much better and happier the population would be! A beginning of a movement has been made in the right direction, under Sir E. Hall's Act. Medical health officers are appointed in the various districts of London, and many of them are working courageously against ignorant opposition, with success. They deserve public approbation, for they have done quietly a great deal of good work, and it is probable, have saved many lives and also prevented much sickness.—*The London Times*

---

*Specialities of the Present Day no Novelty.*—The system of special practice, which is becoming so prevalent at the present time, existed amongst the ancient Egyptians, for Herodotus speaks of their having doctors for almost every part of the body, of which the eye and other organs are particularly mentioned. Our specialism would seem, then, to be merely a revival of an ancient though not enlightened practice.—*London Lancet.*

---

*Death from Chloroform.*—Our readers will find an account of a death from chloroform in this number of our journal. This is the second one that has occurred in this city. The first took place on February 23,1858. We believe this was one of the first deaths reported from inhaling chloroform. Dr. Krause, the gentleman in whose care the recent death took place, is one of the best educated physicians and surgeons in this city or the West. A graduate of one of the German Universities, he has been a respectable practitioner for several years; we can, therefore, say, that no blame is to be attached to him in the case. It is becoming a question, in view of the deaths occurring from chloroform, whether it would not be safer to use a mixture of chloroform and ether. We know the advantages of chloroform, yet we believe that the opinion of practical men who find occasion for its frequent use, will very shortly be established against using it alone.—*Cin. Lan. and Ob.*

---

Lindsay & Blakiston will very soon issue a large work entitled, *American Medical Biography*, by Prof. S. D. Gross. It will consist of memoirs of the most distinguished physicians and surgeons of our country.

*The Memorial to John Hunter.*—It will be recollected by our readers that in closing the vaults of the old church in London, in 1859, Dr. Buckland discovered the remains of the great John Hunter. Great interest was manifested by the learned of all classes; and as a fitting honor to the memory of so great and good a man, his remains were interred in Westminister Abbey, that resting-place of so many of Britain's great men. A subscription was set on foot in England, by the profession, for the purpose of erecting a fitting monument to his memory.

The medical profession of the United States is supposed to entertain an equal veneration for the memory of Hunter with the British profession. At the last meeting of the American medical Association, the following resolution was passed:

*Resolved*, That it be recommended to the different States to collect subscriptions of not more than one dollar each from every regularly educated physician, to aid in the erection of a monument about to be placed in Westminister Abbey to the memory of John Hunter; all moneys collected to be forwarded to the chairman of the committee hereby appointed.—*Cincinnatti Lancet and Observer.*

---

*Remedy for Obesity.*—The use of the leaves and stems of *Tucus vesiculosus*, or common sea-weeds, in decoction, powders, or pills, as a cure of excessive obesity, is strongly advocated by Dr. Duchesne Duparc, in *Champonniere's Jour. of Med. and Surg.*

---

Dr. Meredith Reese will soon put to press "a new and enlarged edition" of his medical lexicon. We are very glad of this, for his dictionary has always been a useful and convenient book to us. We feel sure it will meet with a large sale.—*Cin. Lancet and Observer.*

---

In New Orleans, according to the "N. O. Medical News and Hospital Gazette," there were during the week ending July 15, between seventy-five to eighty cases of sun-stroke. The same journal states that there has not been a single case of yellow fever in that city during the summer, nor one admitted to Charity Hospital.—*Ibd.*

